# Effects of patient-controlled analgesia with hydromorphone or sufentanil on postoperative pulmonary complications in patients undergoing thoracic surgery: a quasi-experimental study

**DOI:** 10.1186/s12871-018-0657-7

**Published:** 2018-12-19

**Authors:** Guangming Yan, Jie Chen, Guiying Yang, Guangyou Duan, Zhiyong Du, Zubin Yu, Jing Peng, Wei Liao, Hong Li

**Affiliations:** 1 Department of Anesthesiology, Anesthesiology of Xinqiao Hospital of Third Military Medical University, Shapingba District, Chongqing, 400037 China; 2 Department of Anesthesiology, Xinqiao Hospital, Shapingba District, Thoracic Surgery of Xinqiao Hospital of Third Military Medical University, Chongqing, 400037 China

**Keywords:** Hydromorphone, Postoperative analgesia, Thoracic surgery, Postoperative pulmonary complications

## Abstract

**Objective:**

To compare the analgesic effects of patient-controlled intravenous analgesia (PCA) with hydromorphone and sufentanil after thoracic surgery on postoperative pulmonary complications (PPCs).

**Methods:**

A total of 142 patients who were scheduled for thoracic surgery were randomly allocated to receive PCA with hydromorphone (group A: experimental group): hydromorphone 0.2 mg/kg + dezocine 0.5 mg/kg + ramosetron 0.6 mg diluted with normal saline to 200 mL; or with sufentanil (group B: control group): sufentanil 3.0μg/kg + dezocine 0.5 mg/kg + ramosetron 0.6 mg diluted with normal saline to 200 mL. The parameters of intravenous analgesia pump were set as background dose 4 ml/h, PCA dose 1 mL, locking time 15 min. Pain NRS (numerical rating scale), Ramsay sedation score, nausea or vomiting score were evaluated at 0 h, 6 h, 12 h, 24 h, 48 h after operation. The cases of PPCs (atelectasis, pulmonary infection, respiratory failure), CRP (C-reaction protein) and inflammatory cells (white cell count and percentage of neutrophils) and blood gas analysis at 12 h after operation, length of ICU and postoperative stay were recorded for each patient.

**Results:**

Data of 136 patients were analyzed. Compared with group B (4[IQR:2,2]), the pain NRS in group A (2[IQR:4,4]) was significantly lower at 6 h after operation (*P* = 0.000). The CRP in group A (69.79 ± 32.13 mg/L) were lower than group B (76.76 ± 43.42 mg/L) after operation, but the difference was not significant (*P* = 0.427). No difference of nausea or vomiting was found between group A (7.3%) and group B (5.8%) postoperatively (*P* = 0.999). The PPCs were happened in 11 patients in group A (16.2%) and 22 patients in group B (32.4%) and the difference between two groups was significant (*P* = 0.027). Seven patients in group A (10.3%) and eighteen patients in group B (26.5%) had clinical evidence of pneumonia and the difference between two groups was significant (*P* = 0.014). The length of ICU and postoperative stay in group A were 2.73 h and 1.82 days less than group B respectively but the differences were not significant (*P* = 0.234, *P* = 0.186 respectively).

**Conclusion:**

Compared with sufentanil, hydromorphone may provide better postoperative analgesic effect with less pulmonary complications for patients undergoing thoracic surgery, and it may accelerate patients’ rehabilitation.

**Trial registration:**

Randomized Controlled Trials ChiCTR1800014282c. Registered 3 January 2018.

## Introduction

For patients undergoing thoracotomy, postoperative pulmonary complications (PPCs) including atelectasis, pulmonary infection and respiratory failure, which were induced by postoperative respiratory dysfunction, were strongly associated with the increase of mortality and postoperative hospital stays, accounting for up to 84% of all deaths [1, 2]. Multiple risk factors are responsible, including the extensive tissue destruction, one lung ventilation and pro-inflammatory cytokines [3]. Patients were not allowed to take deep breath or cough with poor control of postoperation pain and this may lead to atelectasis and retention of secretion [4]. Previous studies have demonstrated that satisfactory postoperative pain relief can enhance patients’ recovery after surgery with better surgical outcomes, less complications and shorter length of hospital stay [5]. So effective management of acute pain after thoracotomy was necessary.

Various strategies had been used for thoracotomy pain management including epidural analgesia, intercostals nerve blockade and systemic opioids like morphine, fentanyl, sufentanil, etc. However, epidural analgesia and intercostals nerve blockade were associated with a considerable risk of sympatholytic complications, misplace, epidural hematoma and abscesses [6–8]. Nowadays, systemic opioids still play an important role in the commonly strategies of patient-controlled intravenous analgesia for thoracic surgery. However, sufentanil can cause some adverse complications such as respiratory depression, which affect safety and recovery of patients [9].

On the other hand, as a potent opioid analgesics, hydromorphone relieves pain through exciting the μ opioid receptor of the central nervous system [10]. It was reported that compared with sufentanil, hydromorphone offered satisfactory postoperative pain therapy with moderate respiratory insufficiency [11, 12]. Therefore, we hypothesized hydromorphone may provide better postoperative analgesia effect with less pulmonary complications for patients undergoing thoracic surgery.

Additionally, multimode Analgesia which combines different methods to enhance the efficiency of analgesic and reduce adverse event is generally accepted. Dezocine was thought as a μ-receptor agonist and a κ-receptor antagonist with a “ceiling effect” for respiratory depression, which could decrease analgesic requirement and attenuate allodynia [13, 14]. The combination of hydromorphone and dezocine may enhance postoperative analgesia after thoracotomy.

Based on the above information, this study aimed to investigate the effects of hydromorphone and sufentanil combining with dezocine on the incidence of PPCs and patient’s outcome.

## Materials and methods

### Patients

After taking institutional ethics committee (The second affiliated hospital, The Third Military Medical University, Chongqing, China) approval, the trial was registered after patient enrollment at Chinese Clinical Trial Registry, http://www.chictr.org.cn/listbycreater.aspx (ChiCTR1800014282c, January 3, 2018). Written informed consent was obtained from all enrolled patients. From December 2017 to March 2018, a total of 142 patients who were scheduled for elective thoracic surgery, aged 20 to 65 years, with American Society of Anesthesiologists (ASA) physical status I or II were recruited. Exclusion criteria included history of severe heart, hepatic or renal disease, pre-existing of lung disease (chronic obstructive pulmonary disease with forced expiratory volume in one second of predicted and/or over forced vital capacity ratio less than 0.8 and/or 0.7 respectively, pneumonia or atelectasis), preoperative respiratory failure (PO_2_<60 mmHg or PCO_2_>50 mmHg), allergy to the studied drugs, and history of chronic pain condition or opioid use.

### Study design

**Fig. 1:**
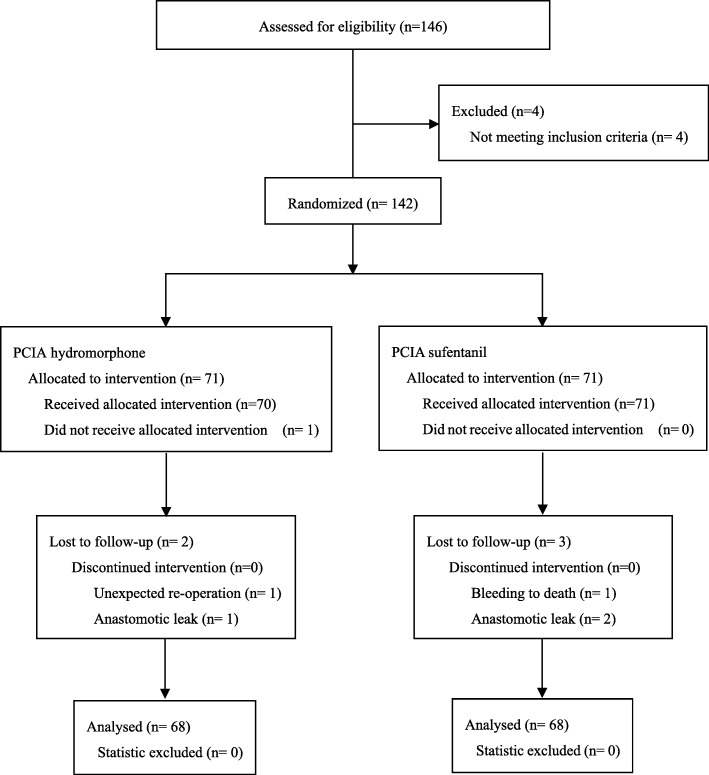
Participant flow. PCIA = patient-controlled intravenous analgesia

On the day before operation, all included patients without premedication were informed the study procedure after written informed consent was obtained. And the use of numerical rating scale (NRS) for evaluation of pain intensity which graduated from 0 (no pain) to 10 (worst pain) was explained to patients. Simple randomization schedule was performed and randomization number was generated using SPSS 19.0 (SPSS Inc., Chicago, IL). The patients were randomly allocated into 2 groups by using opaque sealed envelopes containing the computer-generated randomization schedule (Fig. 1). In the study, patients were blinding to the group allocation. Also, the involved anesthesiologists and surgeons during the surgery and the investigator who performed the postoperative follow-up were not aware of the group allocation. All data and experiment were carried out under the supervision of Data and Safety Monitoring Board.

### Anesthetic procedure

Monitoring of patients during the surgery was accomplished by electrocardiogram (ECG), pulse oximetry (SpO_2_), the radial artery catheter placement, mean arterial pressure (MAP), end-tidal carbon dioxide (_ET_CO_2_) and bispectralindex (BIS). General anesthesia was induced by intravenous injection with midazolam (0.1 mg/kg), atracurium (0.15 mg/kg), propofol (2-3 mg/kg), sufentanil (0.4 μg/kg), and tracheal intubation with a double-lumen tube of appropriate size. Anesthesia was maintained with sevoflurane(1–2%), propofol(6-8 mg/kg/h), remifentanil(0.5-1 μg/kg/min), and atracurium as necessary. Patients were mechanically ventilated with 8 ml/kg during two lung ventilations, reduced to 5 ml/kg with 5 cmH_2_O PEEP during one-lung ventilation and the frequency was adjusted to keep the end tidal carbon dioxide (ETCO_2_) between 35 and 45 mmHg [15]. The inspired oxygen fraction (FiO_2_) was increased if necessary to maintain oxygen saturations (SPO_2_) greater than 90%. Alveolar recruitment strategy: the PEEP was increased from 5 cmH_2_O to 40 cmH_2_O by a level of 5 cmH_2_O at a ventilatory frequency of 8 bpm with a tidal volume of 7–8 ml/kg until the alveolar recruited, and then reduced to previous 5 cmH_2_O gradually [16].

For all included patients, operations were done by the same experienced surgeons. After the end of the skin closure, all patients were given sufentanil (0.1 μg/kg), Ramosetron (0.3 mg), and if patients reported the pain-NRS ≥ 4, followed by 5 mg boluses dezocine.

Postoperative pain management.

Mechanical intravenous analgesia pump (200 mL, Beijing KSH Technology Institute, Beijing, China) was used and parameters were set as background dose 4 ml/h, PCA dose 1 ml, locking time 15 min. In the hydromorphone (HUMANWELL HEALTHCARE, Hubei, China) group (group A) PCA with a mixture of hydromorphone (0.2 mg/kg), dezocine (0.5 mg/kg, Yangtze River Pharmaceutical, China), and ramosetron 0.6 mg was applied. While in sufentanil (HUMANWELL HEALTHCARE, Hubei, China) group (group B) PCA with a compound of sufentanil (3.0μg/kg), dezocine (0.5 mg/kg), and ramosetron (0.6 mg) was used. During the PCA treatment, when inadequate analgesia presented, rescue analgesia was given suing dezocine 5 mg.

Outcomes.

PPCs evaluation was performed by an independent and experienced surgeon after surgery at the same time each day up to discharge. Using the Melbourne Group Scale (MGS), PPCs was defined in those patients presenting with four or more of the following eight dichotomous factors: temperature>38 °C; white cell count>11.2 × 10^9^/L; chest X-ray findings of atelectasis or consolidation; signs of infection on sputum microbiology; purulent sputum; physician diagnosis of pneumonia; SPO2<90% on air; and prolonged intensive care (ICU) or hospital stay [17]. Respiratory failure was defined according to artery gas analysis (PO_2_<60 mmHg or PCO_2_>50 mmHg) when patients respired air [18].

Rest pain NRS was considered as the secondary outcome. In addition, demographic data including age, weight, smoking index (the number of cigarettes smoked per day multiply by number of years of smoking) [19], and artery blood gas analysis were recorded before operation. Intraoperatively, duration of surgery, total dose of remifentanil and sufentanil were recorded. Pain NRS (0, no pain to 10, worst pain), Ramsay sedation score (1, anxious, agitated, or restless; 2, cooperative, oriented, and tranquil; 3, response to command; 4, brisk response; 5, a sluggish response; 6, no response) and nausea or vomiting score (0, without nausea or vomiting; 1, mild; 2, middle; 3, serious) were collected at 0 h, 6 h, 12 h, 24 h, 48 h after extubation [20]. The skin pruritus was divided as mild, middle and server. CRP (C-reaction protein), inflammatory cells and blood gas analysis at 12 h after operation were also measured. And length of ICU and postoperative stay was recorded.

### Statistical analysis

The primary outcome of the current study was the incidence of PPCs. Previously published data have showed that the incidence of PPCs was 28% [21]. On the basis of preliminary experimental data, we hypothesized hydromorphone may reduce the odds of pulmonary complications to 15%. Under these conditions, 56 patients per group were required to reach a power of 80% (one-side hypothesis) and a 0.05 risk of type I error. Thus, considering about 20% loss of follow-up, we decided to include 71 evaluable patients for each group.

Date were presented as number, percentages, median (range) or mean ± SD and analyzed using the SPSS 19.0 software. Chi-square test was used to compare the difference of categorical data between two groups. The difference of pain NRS between two groups was compared by the use of two independent samples nonparametric test (the Mann-Whitney test). Independent samples t-test was used to compare the continuous variable data between 2 groups. Two side *P*-value < 0.05 was considered to be statistically significant.

## Result

### General results

After exclusion of one patient (the patient in the hydromorphone group because of failure to receive allocated intervention), 141 patients were considered of analysis. Then, five patients were excluded for unexpected re operation, anastomotic leak or bleeding to death (Fig. 1). Thus, the pain NRS and PPCs analyses were based on 68 patients in the hydromorphone group and 68 patients in the sufentanil group.

As shown in Table 1, no difference was found in age, height, weight, gender, smoking index, surgery types, preoperative inflammatory cells and blood gas analysis, intraoperative dose of remifentanil and sufentanil, and duration of the surgery between patients in group A and group B (*P* > 0.05).

**Table 1:** Demographic data and patients’ characteristics

	Group A (*n* = 68)	Group B (n = 68)	*P-*value
Age (yr)	51.03 ± 10.02	48.37 ± 11.09	0.144
Height (cm)	162.74 ± 7.35	163.83 ± 9.11	0.442
Gender (F/M)	29/39	27/41	0.727
Weight (kg)	61.35 ± 7.64	60.69 ± 8.08	0.621
Smoking index	197.94 ± 349.10	207.35 ± 281.83	0.863
Duration of surgery (min)	178.51 ± 75.22	179.69 ± 68.08	0.924
Total amount of sufentanil (μg)	62.97 ± 14.16	60.59 ± 13.86	0.323
Total amount of remifentanil (mg)	1.18 ± 0.60	1.29 ± 0.68	0.329
Type of surgery			1.000
Thoracoscopic	54	54	
Open Esophagectomy	14	14	
Total amount of fluid use (mL)	1559.85 ± 588.93	1538.53 ± 512.87	0.891
White cell count (10^9^/L)	6.00 ± 2.20	6.18 ± 2.21	0.643
Percentage of neutrophils (%)	61.81 ± 10.05	60.08 ± 10.67	0.340
PH	7.42 ± 0.05	7.43 ± 0.03	0.202
PO_2_ (mmHg)	85.38 ± 11.15	84.45 ± 12.36	0.648
PCO_2_ (mmHg)	40.63 ± 4.31	40.46 ± 3.52	0.808

Smoking index = the number of cigarettes smoker per day multiply by number of years of smoking. Total amount of fluid use = Fluid therapy during surgery (crystalloid and colloid). Data given as mean ± SD or number of patients

### Postoperative analgesic effects and consumption of analgesics

**Fig. 2:**
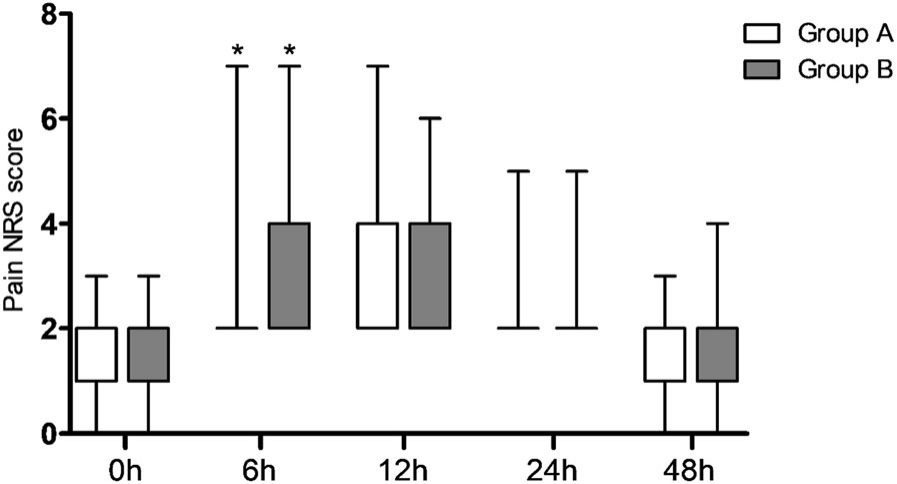
Pain NRS (numerical rating scale) distribution in both the groups at 0 h(A), 6 h(B), 12 h(C), 24(D)and 48 h(E) after surgery. (* represented significant difference between group A and group B)

Pain NRS and Ramsay sedation scores were measured at 0 h, 6 h, 12 h, 24 h, and 48 h after the surgery. The results showed that pain NRS at 6 h after the surgery in group A was significantly higher than that in group B (*P* < 0.05), and there was no difference in pain NRS at other time points between two groups (*P* > 0.05, Fig. 2). No significant difference of Ramsay sedation score was observed between two groups (*P* > 0.05, Table 2). In addition, there was no significant difference in consumption of analgesics for patients during PCA between two groups (*P* > 0.05, Table 3).

**Table 2:** Ramsay sedation scores at 0 h, 6 h, 12 h, 24 h, and 48 h after the surgery

	0 h				6 h				12 h				24 h				48 h		
	0	1	2		0	1	2		0	1	2		0	1	2		0	1	2
Group A (n = 68)	66	2	0		65	1	2		66	2	0		68	0	0		68	0	0
Group B (n = 68)	67	1	0		68	0	0		67	0	1		67	1	0		68	0	0
*P* value	0.999				0.215				0.222				0.999				1.000

**Table 3:** The consumption of analgesics at 1d, and 2d after the surgery

	Group A (n = 68)	Group B (*n* = 68)	*P* value
1d (mL)	100.74 ± 5.52	101.20 ± 6.72	0.697
2d (mL)	95.74 ± 3.93	95.00 ± 4.75	0.380

### Adverse events assessment

The side-effects of nausea or vomiting were complained mainly at 6 h and 12 after surgery, but the difference between two groups was not significant at all time points postoperatively(*P* > 0.05, Table 4). In addition, skin pruritus was not observed in all patients.

**Table 4:** nausea or vomiting scores at 0 h, 6 h, 12 h, 24 h, and 48 h after the surgery

	0 h				6 h				12 h				24 h				48 h		
	0	1	2		0	1	2		0	1	2		0	1	2		0	1	2
Group A (n = 68)	68	0	0		65	2	1		66	1	1		67	1	0		68	0	0
Group B (n = 68)	68	0	0		66	1	1		66	2	0		68	0	0		68	0	0
*P* value	1.000				0.843				0.312				0.999				1.000		

### Plasma CRP and blood gas analysis

Analysis of variance showed that plasma CRP level in group B was not significantly higher than that in group A (*P* > 0.05, Table 5). There was no significant difference between two groups in White cell count, Percentage of neutrophils, pH, PO_2_ (mmHg), PCO_2_ (mmHg) and BE value at 12 h after operation (*P* > 0.05, Table 5).

**Table 5:** Plasma CRP and blood gas analysis at 12 h after operation

	Group A (n = 68)	Group B (n = 68)	*P* value
CRP (mg/L)	69.79 ± 32.13	76.76 ± 43.42	0.427
White cell count (10^9^/L)	9.49 ± 2.83	10.55 ± 3.43	0.056
Percentage of neutrophils (%)	75.94 ± 8.38	78.50 ± 6.54	0.051
PH	7.39 ± 0.03	7.40 ± 0.04	0.377
PO_2_ (mmHg)	114.15 ± 26.93	113.75 ± 36.02	0.942
PCO_2_ (mmHg)	43.09 ± 4.35	42.58 ± 7.52	0.509
BE	1.61 ± 2.52	2.41 ± 2.38	0.062

*CRP* C-reactive protein

### Primary outcomes

The incidence of PPCs including pneumonia, atelectasis and respiratory failure in group A was lower than group B, and the difference between two groups was significant (*P* = 0.027, Table 6). The length of ICU and postoperative stay in group A were less than group B respectively but the differences were not statistically significant (*P*>0.05, Table 7).

**Table 6:** primary outcomes

	Group A (n = 68)	Group B (n = 68)	*P* value
pneumonia	7(10.3%)	18(26.5%)	0.014
atelectasis	8(11.8%)	6(8.8%)	0.572
respiratory failure	0(0%)	1(1.5%)	0.315
Pneumonia and atelectasis	4(5.9%)	3(4.4%)	0.697
Number of PPCs	11(16.2%)	22(32.4%)	0.027

*PPCs* postoperative pulmonary complications (pneumonia, atelectasis and respiratory failure)

**Table 7:** The ICU stay and length of postoperative stay

	Group A (n = 68)	Group B (n = 68)	*P* value
ICU stay (h)	57.75 ± 13.71	60.48 ± 12.96	0.234
Postoperative stay(d)	9.06 ± 3.38	10.88 ± 7.17	0.186

*ICU* intensive care unit

## Discussion

This study used a double blind randomized controlled design to investigate the efficacy of PCA with hydromorphone in the prevention of postoperative pulmonary complication compared with sufentanil. The results showed that hydromorphone may provide better postoperative analgesia and anti-inflammatory effect with less pulmonary complications and accelerate patients’ rehabilitation for patients undergoing thoracic surgery.

By stimulating the μ opioid receptor of the central nervous system, hydromorphone played an important role in analgesia, especially in acute pain treatment [5, 10]. The equi-analgesic ratio for morphine to hydromorphone was reported between 5:1–7:1 [22]. Sufentanil was often reported to be about 400–1000 times more than morphine [23]. Chun-Shan Dong et al. reported that 3.0 μg/kg sufentanil can improve pain control after thoracotomy [24]. Therefor choosing the appropriate potency ratio (50:1), hydromorphone 0.2 mg/kg can be considered equipotent to sufentanil 3.0 μg/kg.

Compared to group sufentanil, the pain NRS at 6 h after operation in group hydromorphone was significantly lower. The median protein binding of hydromorphone is 11.6% with the free fraction remaining nearly constant, whereas the protein binding of sufentanil was 88.4% with the free fraction increasing towards the end of the PCA period [12]. Thus, in the early period of the PCA, the analgesic effect of hydromorphone was better than sufentanil.

The level of CRP (C-reactive protein) in plasma increases greatly during acute phase response to tissue injury, infection, or other inflammatory stimuli [25]. Khaled M. Fares et al. found that pro-inflammatory cytokines increased to their zenith at 1 h after Esophagectomy [21].There were significant inverse correlations between pain intensity and the plasma inflammatory cytokines concentrations [26]. Comparing with group sufentanil, the CRP was lower in group hydromorphone. One possible explanation of this was that the analgesic effect of hydromorphone was better than sufentanil in the early period of the PCA. But the difference between the two groups was not significant. Carvalho, B et al. reported that hydromorphone cannot reduce wound exudate concentrations of interleukin-6 and interleukin-10 [27]. Further research with more inflammatory cytokines measurement was necessary.

The major cause of postoperative morbidity and mortality after thoracotomy is PPCs, and inadequate postoperative analgesia can result in splinting, retention of secretions and atelectasis and further may compromise the respiratory functions [1]. Pulmonary function was severely decreased to 39% of the basic line on the first day and rehabilitated gradually [28]. The incidence of PPCs may be reduced by a better postoperative analgesia with a lower respiratory depression [29]. Jeleazcov, C et al. found that PCA with hydromorphone offered satisfactory postoperative analgesic with respiratory insufficiency in 5% of the patients [11]. Correspondingly, Deng, C et al. had reported that the risks of respiratory depression in patients undergoing colonoscopy was 33% by using sufentanil as a perioperative analgesia [30]. As mentioned above, hydromorphone also had a quicker therapeutic and better anti-inflammatory effect comparing with sufentanil. So, our results of postoperative pulmonary complications were consistent with this explanation. With respect to outcomes, we observed a significant decrease of pneumonia and a better trend of length of ICU and postoperative stay in group hydromorphone, even if the difference of length of ICU and postoperative stay between both groups was statistically insignificant.

Dezocine was regard as a partial μ-receptor agonist, a κ-receptor antagonist, and a norepinephrine and serotonin reuptake inhibitor [31]. Recently another research suggested that dezocine can attenuate allodynia by spinal μ-opioid receptor antagonism or norepinephrine depletion/α2-adrenoceptor antagonism [14]. A Meta-Analysis of Randomized Controlled Trials suggested that Dezocine was a promising analgesic for preventing postoperative pain [32]. Wang, C et al. had reported that combined dezocine and sufentanil might be a complement drug for sufentanil in PCA with limited side effects [19]. Another research reports that sufentanil may increase the contractile tension of intestine smooth muscle, while dezocine does not [33]. Our study indicated that dezocine can be safely used with hydromorphone in PCA after thoracotomy without increasing the adverse reaction.

### Study limitations

The different surgical trauma and combined application of various analgesics may become potential factors that interfere with the results of our study. And also, the insignificant difference of the length of ICU and postoperative stay may be limited by its sample size. Concerning of the limitations of our study, further innovative strategies will be required to investigate the specific effect of hydromorphone on lung function.

## Conclusion

In conclusion, we have found that the pain relief in the hydromorphone group was observed to result in better outcomes including lesser PPCs, and shorter length of the ICU and postoperative stay. Thus, hydromorphone may be suitable opioid to patient-controlled intravenous analgesia for patients undergoing thoracotomy.

## Abbreviations

ASA: American Society of Anesthesiologists
BIS: Bispectralindex
CRP: C-reaction protein
ICU: Intensive care unit
MGS: Melbourne Group Scale
NRS: Numerical rating scale
PCA: Patient-controlled intravenous analgesia
PEEP: Positive end-expiratory pressure.
PPCs: Postoperative pulmonary complications
